# Natural Killer Cells and Host Defense Against Human Rhinoviruses Is Partially Dependent on Type I IFN Signaling

**DOI:** 10.3389/fcimb.2020.510619

**Published:** 2020-10-21

**Authors:** Saskia L. van der Heide, Yang Xi, John W. Upham

**Affiliations:** ^1^ Lung and Allergy Research Centre, Diamantina Institute, The University of Queensland, Woolloongabba, QLD, Australia; ^2^ Department of Respiratory Medicine, Princess Alexandra Hospital, Brisbane, QLD, Australia

**Keywords:** natural killer cell, rhinovirus, type I interferon, antiviral immunity, cytokine production, degranulation

## Abstract

Rhinovirus (RV), the causative agent of the common cold, causes only mild upper respiratory tract infections in healthy individuals, but can cause longer lasting and more severe pulmonary infections in people with chronic lung diseases and in the setting of immune suppression or immune deficiency. RV-infected lung structural cells release type I interferon (IFN-I), initiating the immune response, leading to protection against viruses in conjunction with migratory immune cells. However, IFN-I release is deficient in some people with asthma. Innate immune cells, such as natural killer (NK) cells, are proposed to play major roles in the control of viral infections, and may contribute to exacerbations of chronic lung diseases, such as asthma. In this study, we characterized the NK cell response to RV infection using an *in vitro* model of infection in healthy individuals, and determined the extent to which IFN-I signaling mediates this response. The results indicate that RV stimulation *in vitro* induces NK cell activation in healthy donors, leading to degranulation and the release of cytotoxic mediators and cytokines. IFN-I signaling was partly responsible for NK cell activation and functional responses to RV. Overall, our findings suggest the involvement of NK cells in the control of RV infection in healthy individuals. Further understanding of NK cell regulation may deepen our understanding of the mechanisms that contribute to susceptibility to RV infections in asthma and other chronic lung diseases.

## Introduction

Respiratory viruses, particularly rhinoviruses (RV), typically cause only mild, self-limited infections in healthy individuals, but the consequences of infection can be much more serious in people with chronic lung diseases and in the setting of immune suppression or immune deficiency (Glezen et al., 2000; Versluys et al., 2010; Costa et al., 2011; Jacobs et al., 2013). For example, RV are implicated in both the induction of asthma and exacerbations of established asthma, representing the most common cause of asthma-related death (Longini et al., 1984; Beasley et al., 1988; Gern et al., 1996; Papadopoulos et al., 2002a; Papadopoulos et al., 2002b; Hansbro et al., 2008). Asthmatics are no more likely than healthy individuals to develop a cold, but are substantially more likely for the infection to spread to the lower respiratory tract, causing more severe symptoms (Corne et al., 2002). This raises important questions regarding the mechanisms by which healthy people are able to mount an effective host response to RV. Understanding these processes that occur in healthy individuals might help to explain why RV infections can have such severe outcomes in people with asthma.

Bronchial epithelial cells (BECs) are the primary point of contact between infecting respiratory viruses and the host (Bals and Hiemstra, 2004). During infections, BECs release type I (IFN-I) and III IFNs, which are integral for the induction of innate immune response, conferring protection against viruses in conjunction with migratory immune cells (Hansbro et al., 2008; Message et al., 2008). Some investigators report that RV-induced IFN-I release is deficient in asthma (Gern et al., 2000; Corne et al., 2002; Wark et al., 2005; Hansbro et al., 2008), though other reports have been unable to confirm these findings (Sykes et al., 2014; Xi et al., 2015). Cell-mediated immunity to RV is also important in the control and clearance of infection and may be abnormal in asthmatics (Message and Johnston, 2001). Innate immune cells, such as natural killer (NK) cells have been proposed to play a major role in both asthma (Message et al., 2008; Culley, 2009), and the control of viral infections in general (Biron et al., 1999; Biron and Brossay, 2001; Barnig et al., 2013; Lam and Lanier, 2017); however, little is known about the role NK cells play in RV infections.

NK cells are large, granular lymphocytes involved in the innate immune response to infected or malignant cells (Trinchieri, 1989; Alter et al., 2004; Vivier et al., 2008; Bryceson et al., 2010; Montaldo et al., 2013). Without prior exposure or antigen sensitisation, NK cells are able to detect and kill infected or aberrant cells, and produce chemokines and cytokines that prime and shape adaptive immunity (Kay, 1985; Colucci et al., 2003; French and Yokoyama, 2003; Bryceson et al., 2010). Interestingly, circulating NK cells appear to reflect the maturation status of lung-resident NK cells, with a phenotype capable of both cytokine production and cytotoxicity (Ivanova et al., 2014).

Human NK cells are identified as CD56^+^ lymphocytes that lack markers specific to other lymphocytes, such as CD3 on T cells, CD14 on monocytes, and CD19 on B cells (Herberman and Ortaldo, 1981; Robertson and Ritz, 1990; Bancroft, 1993; D’Arena et al., 1998; Papamichail et al., 2004). Two distinct subsets of human NK cells have been identified and classified based on the cell-surface density of CD56; these subsets appear to have distinct phenotypic and functional properties (Lanier et al., 1986; Cooper et al., 2001a; Sedlmayr et al., 2004; Moretta, 2010). The CD56^dim^ subset of NK cells appear more naturally cytotoxic, but have a reduced ability to produce cytokines upon stimulation (Cooper et al., 2001a; Di Santo, 2008); while the CD56^bright^ subset of NK cells are distinguished by potent production of both Th1 and Th2 cytokines in response to stimulation, but have reduced cytotoxic capacity (Lanier et al., 1986; Cooper et al., 2001b).

NK cell recognition of target cells relies on an assortment of both activating and inhibiting receptors that detect microbial products, cytokines, stress signals, and MHC class I molecules (Lanier, 2000; Smyth et al., 2000; Moretta et al., 2001; Radaev and Sun, 2003). NK cell activation does not rely on an individual receptor, but on a combination of activating and inhibiting signals, with activation occurring when different combinations of receptors are ligated to overcome a threshold of activation (Bryceson et al., 2005; Lanier, 2008). NK cell surface receptor repertoire has been reported to vary between subsets, and thus subset activation may vary based on the stimulus (Cooper et al., 2001a; Sedlmayr et al., 2004).

NK cell activation can also be induced by interactions with antigen-presenting cells (APCs), such as DCs and macrophages, and by cytokines, including IFN-I (Gern et al., 1996; Biron et al., 1999; Biron and Brossay, 2001; Moretta, 2002; Vivier et al., 2008). Increased cell-surface CD69 expression is widely used as a marker of NK cell activation (Gern et al., 1996; Vering et al., 2000; Draghi et al., 2007; Barnig et al., 2013).

NK cells can lyse target cells through the directed exocytosis of cytolytic granules containing perforin and granzyme at the immunological synapse between the NK cell and target cell (Robertson and Ritz, 1990; Cooper et al., 2001a; Artis and Spits, 2015). In particular, granzyme B (GzymB) has been shown to be required for the rapid induction of NK cell-mediated apoptosis of target cells (Shresta et al., 1995). The release of these cytolytic granules can be indicated through the presence of cell surface CD107a (Alter et al., 2004; Bryceson et al., 2010).

In addition to cytolytic function, NK cells produce chemokines and cytokines that can recruit other immune cells, promote cellular resistance to infection, and influence the formation of adaptive immunity (Robertson and Ritz, 1990; Bancroft, 1993; Cooper et al., 2001a; Bryceson et al., 2010). An important cytokine produced by NK cells is IFNγ, with known immunoregulatory effects (Gern et al., 1996; Cooper et al., 2001a; Cooper et al., 2001b; Colucci et al., 2003; Maroof et al., 2008; Strowig et al., 2008; Bi et al., 2017).

With the strong association between respiratory viral infections and asthma exacerbations, it has been speculated that inappropriate immune activation, possibly due to defective IFN-I response, may be involved in the susceptibility of asthmatics to more persistent and severe infections (Wark et al., 2005; Message et al., 2008; Culley, 2009).

Therefore, this study aimed to determine whether RV can activate NK cells, and the extent to which NK cell activation and function *in vitro* are IFN-I dependent.

## Materials and Methods

### Participants

All volunteers completed a detailed questionnaire regarding respiratory symptoms, prior medical diagnoses, and medication use. Healthy participants had no symptoms or prior self-reported physician diagnoses of respiratory disease (including asthma) and had not experienced respiratory infection symptoms within the preceding month. All participants underwent skin prick testing (SPT) against a panel of common allergens (*Aspergillus fumigatus*, Alternaria, grass pollen, house dust mite, and dog and cat dander) to determine allergic status. Prior to sample collection, volunteers were asked to abstain from antihistamine use for 72 h. The study was approved by the Human Ethics Committees at The University of Queensland and the Princess Alexandra Hospital, and all participants provided written consent.

### Rhinovirus Generation and Titration

RV16 stocks were generated by passage in Ohio HeLa cells, as previously described (Sanders et al., 1998). RV16 was purified over Optiprep gradient (Sigma-Aldrich) as described by Xi et al. (2015). Viral titer was determined using TCID50, and RV16 at an MOI of 1 was used in culture (Pritchard et al., 2012).

### PBMC Isolation and Culture

Human peripheral blood was collected into heparinized tubes (BD Vacutainer) from 12 healthy volunteers (mean age 21.6 ± 2.8 years, 67% female, 25% atopic). Peripheral blood mononuclear cells (PBMCs) and plasma were isolated by density gradient centrifugation with Lymphoprep (Stemcell Technologies). PBMC suspensions (4×10^6^ cells/ml) were prepared in media (RPMI 1640 (Gibco) supplemented with 5% autologous plasma, 2-ME, penicillin, streptomycin, and glutamine). 500 µL of the PBMC suspensions (2×10^6^ cells) were seeded on a 24-well flat bottom plate. IFN-I signaling was blocked in PBMC cultures using recombinant B18R protein (100 ng/ml, eBioscience), alongside a media-only control, for 1 h at 37°C with 5% CO_2_. B18R acts as a decoy receptor for IFN-I with high specificity and affinity for all known subtypes of the IFN-I family, blocking IFN-I signaling into target cells (Colamonici et al., 1995; Symons et al., 1995; Alcamí et al., 2000; Pritchard et al., 2012; Pritchard et al., 2014). PBMC cultures were then stimulated with RV16 (MOI=1), or media alone, for 24 h at 37°C with 5% CO_2_. To assess NK cell degranulation, CD107a-BV786 Ab (**Table S1**) was added for the last 6 h of culture. Brefeldin A (BFA, BioLegend) and monensin (GolgiStop™, BD Biosciences) were added for the last 5 h of cultures to allow for the detection of intracellular cytokine production and the detection of reinternalized CD107a, respectively, as previously described (Alter et al., 2004).

### Flow Cytometry

Flow cytometry was used to identify immune cell populations and their expression of activation and function markers. Cell cultures were centrifuged, cell pellets were washed with PBS and labeled with live/dead (L/D) aqua viability dye (1:500 in PBS, Thermo Fisher Scientific) for 30 min on ice, protected from light, to allow for the exclusion of dead cells. Cells were surface stained with the following Abs: CD3-FITC, CD14-PerCp-Cy5.5, CD19-APC, CD56-PE-Cy7, and CD69-APC-Cy7 (**Table S1**); for 30 min on ice, protected from light. Cell were then fixed and permeabilised using the Cytofix/Cytoperm™ Fixation/Permeabilisation kit (BD Biosciences) and stained intracellularly with IFNγ-PE and GzymB-BV421 Abs (**Table S1**) for 30 min at room temperature, protected from light. Stained cells were then fixed in 2% paraformaldehyde. A total of ~200,000 events per sample were collected using LSRFortessa X-20 (BD Biosciences) and the results were analyzed using FlowJo software (v10.7, Tree Star, Inc.). NK cells were identified as CD3^–^CD14^–^CD19^–^CD56^+^ lymphocytes, activated NK cells as CD3^–^CD14^–^CD19^–^CD56^+^CD69^+^, and degranulating NK cells as CD3^–^CD14^–^CD19^–^CD56^+^CD107a^+^ (Herberman and Ortaldo, 1981; Robertson and Ritz, 1990; Bancroft, 1993; D’Arena et al., 1998; Vering et al., 2000; Alter et al., 2004; Papamichail et al., 2004; Draghi et al., 2007; Bryceson et al., 2010; Wang et al., 2012; Barnig et al., 2013).

### ELISA

Cell-free culture supernatants were collected and stored in deep-well plates at −30°C, until required. IFNγ and GzymB concentrations in culture supernatant were measured using ELISA. IFNγ assays used commercially available paired Abs and recombinant cytokines (BD Pharmingen; limit of detection = 3.91 pg/ml). GzymB assays used a commercially available ELISA kit (R&D Systems; limit of detection = 39.1 pg/ml), according to the manufacturer’s instructions.

### Statistical Analysis

Data were analyzed using Graphpad Prism (version 8.1) using Wilcoxon matched-pairs signed rank tests to compare differences between paired samples. Data are represented as median (interquartile range – IQR). A p value of <0.05 was considered statistically significant.

## Results

### RV16, But Not IFN-I Signaling, Causes Minor Changes in NK Cell Populations

To determine whether RV could activate NK cells, and the extent to which NK cell activation and functional changes are IFN-I dependent, PBMCs from healthy volunteers were cultured for 1 h in the presence or absence of B18R to block IFN-I signaling, prior to culture for 24 h in the presence or absence of RV16 stimulation. Following this, flow cytometry was used to identify and evaluate the frequency of CD56^+^ NK cells, along with CD56^dim^ and CD56^bright^ NK cell subsets (**Figure S1**).

**Figure 1:**
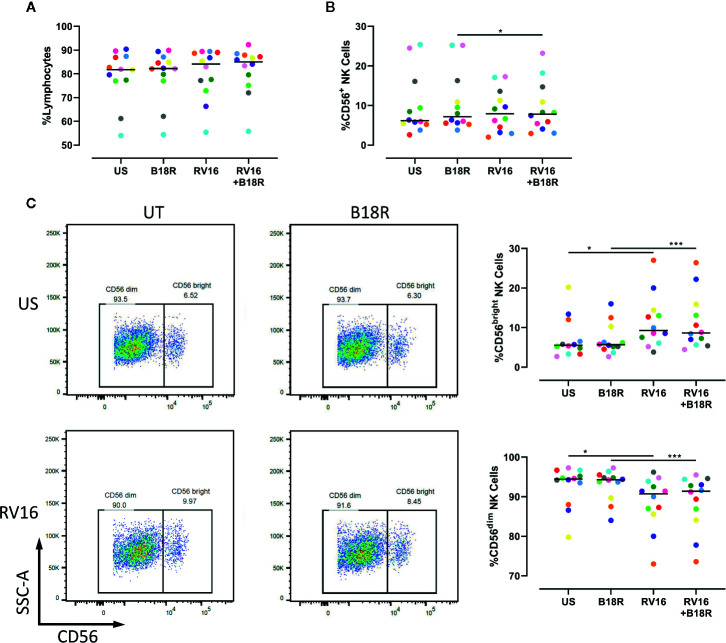
RV16 altered NK cell populations, in an IFN-I independent manner. PBMCs from healthy people (n=12) were cultured *in vitro* with B18R (100 ng/ml) for 1 h to block IFN-I signaling, alongside a media-only control (UT), prior to stimulation with RV16 (MOI = 1), alongside an unstimulated control (US) for 24 h. **(A)** Percentage of lymphocytes, **(B)** total CD56^+^ NK cells, **(C)** and NK cell subsets (CD56^dim^ and CD56^bright^) were evaluated using flow cytometry. Raw dot plots are representative of all 12 healthy donors. Each colored symbol represents data from one donor, lines represent medians. Data are representative of three experiments. *p<0.05, ***p<0.001 by Wilcoxon matched-pairs signed rank tests. RV16, rhinovirus 16; IFN-I, type I interferon; NK, natural killer; PBMC, peripheral blood mononuclear cell; UT, untreated; MOI, multiplicity of infection; US, unstimulated; SSC-A, side scatter-area.

Our results demonstrate that neither RV16 stimulation, nor blocking of IFN-I signaling, altered the frequency of lymphocytes (**Figure 1A**), though RV16 induced a small increase in CD56^+^ NK cell frequency that was less apparent when IFN-I signaling was blocked (**Figure 1B**). Stimulation with RV16 induced minor changes in the distribution of CD56^dim^ and CD56^bright^ NK cell subsets, with a decrease in the frequency of CD56^dim^ NK cells and a corresponding increase in the frequency of CD56^bright^ NK cells (**Figure 1C**). These changes in the distribution of the CD56^dim^ and CD56^bright^ NK cell subsets were not substantially altered when IFN-I signaling was blocked.

### RV16 Induces Intense NK Cell Activation, Which Is Partly Dependent on IFN-I Signaling

**Figure 2:**
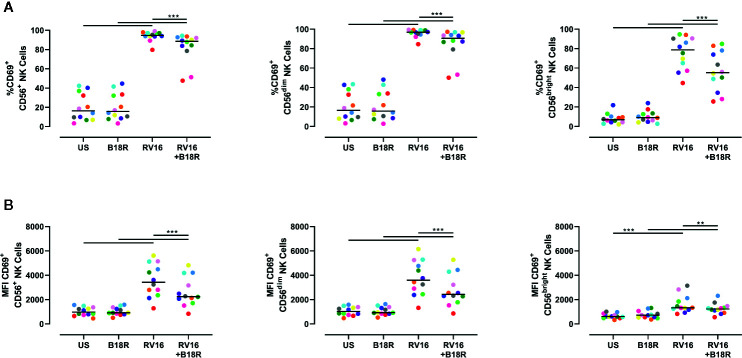
RV16 induces NK cell activation as assessed by CD69 expression, and this is attenuated by blocking of IFN-I signaling. PBMCs from healthy people (n=12) were cultured *in vitro* with B18R (100 ng/ml) for 1 h, prior to stimulation with RV16 (MOI = 1), alongside an unstimulated control (US) for 24 h. **(A)** Percentage of activated (CD69+) CD56^+^ (left), CD56^dim^ (middle), and CD56^bright^ (right) NK cells. **(B)** Level of expression (indicated by MFI) of the activation marker (CD69) on CD56^+^ (left), CD56^dim^ (middle), and CD56^bright^ (right) NK cells. Each colored symbol represents data from one donor, lines represent medians. Data are representative of three experiments. **p<0.01, ***p<0.001 by Wilcoxon matched-pairs signed rank tests. IFN-I, type I interferon; NK, natural killer; RV16, rhinovirus 16; PBMC, peripheral blood mononuclear cell; UT, untreated; MOI, multiplicity of infection; US, unstimulated; MFI, median fluorescence intensity.

NK cell activation was assessed based on cell surface CD69 expression. Both an increase in the frequency of CD69^+^ cells and the expression intensity of CD69 can be used to assess NK cell activation (Draghi et al., 2007; Du et al., 2010; Souza-Fonseca-Guimaraes et al., 2012; Barnig et al., 2013). RV16 stimulation of PBMC for 24 h led to substantial and significant increases in the proportion of NK cells expressing CD69, though this occurred to a lesser extent in the absence of IFN-I signaling (**Figure 2A**, left). Blocking IFN-I signaling had a larger impact on the percentage of CD69^+^ cells in the CD56^bright^ subset (**Figure 2A**, right) than in the CD56^dim^ subset (**Figure 2A**, middle). RV16 also increased the median fluorescent intensity (MFI) of CD69 surface expression on NK cells (**Figure 2B**), especially the CD56^dim^ subset (**Figure 2B**, middle).

### RV16 Induces NK Cell Cytolytic Granule Release Which Is Partly Dependent on IFN-I Signaling

**Figure 3:**
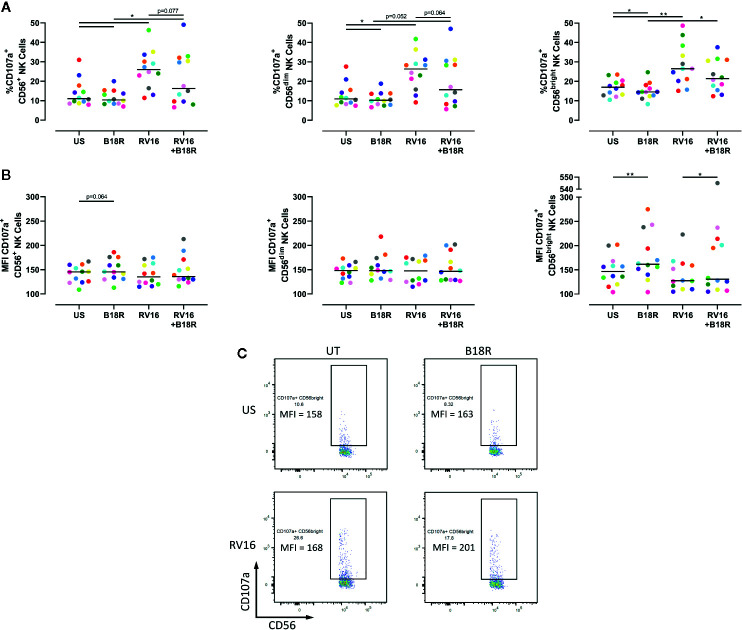
RV16 stimulation enhances NK cell CD107a expression, and this is attenuated by blocking of IFN-I signaling. PBMCs from healthy people (n=12) were cultured *in vitro* with B18R (100 ng/ml) for 1 h, alongside a media-only control (UT), prior to stimulation with RV16 (MOI = 1), alongside an unstimulated control (US) for 24 h. **(A)** Percentage of degranulating (CD107a+) CD56^+^ (left), CD56^dim^ (middle), and CD56^bright^ (right) NK cells. **(B)** Level of surface expression (indicated by MFI) of the degranulation marker (CD107a) on CD56^+^ (left), CD56^dim^ (middle), and CD56^bright^ (right) NK cells. **(C)** Frequency and MFI of CD107a^+^ CD56^bright^ NK cells. Each colored symbol represents data from one donor, lines represent medians. Data are representative of three experiments. *p<0.05, **p<0.01 by Wilcoxon matched-pairs signed rank tests. IFN-I, type I interferon; NK, natural killer; RV16, rhinovirus 16; PBMC, peripheral blood mononuclear cell; UT, untreated; MOI, multiplicity of infection; US, unstimulated; MFI, median fluorescence intensity.

NK cell degranulation was assessed based on CD107a surface expression. CD107a lines the cytolytic granules that are secreted during cytolysis, and appearance at the cell surface is upregulated following stimulation, correlating with target cell lysis (Alter et al., 2004; Bryceson et al., 2005; Bryceson et al., 2010). The presence of CD107a at the cell surface is an indicator of NK cell release of cytotoxic granules (Haworth et al., 2011; Barnig et al., 2013). RV16 stimulation of PBMC cultures resulted in significant increases in the proportion of CD56^+^ NK cells expressing cell surface CD107a (**Figure 3A**, left). Blocking of IFN-I signaling *in vitro* led to a lower frequency of degranulating CD56^+^ NK cells, both in the presence and absence of RV16 stimulation. These trends were also observed in both the CD56^dim^ (**Figure 3A**, middle) and CD56^bright^ NK cell subsets (**Figure 3A**, right). Stimulation with RV16 had no significant effect on the MFI of CD107a surface expression on CD56^+^ NK cells (**Figure 3B**, left). There were no significant changes in the level of CD107a surface expression in the CD56^dim^ NK cell population (**Figure 3B**, middle). However, in the CD56^bright^ NK cell population (**Figure 3B**, right), blocking of IFN-I signaling resulted in a small increase in the MFI of CD107a surface expression, in both the presence and absence of RV16 stimulation (**Figure 3C**).

### RV16 Induces Small Changes in Both the Percentage of GzymB-Producing NK Cells and Their Intracellular GzymB

**Figure 4:**
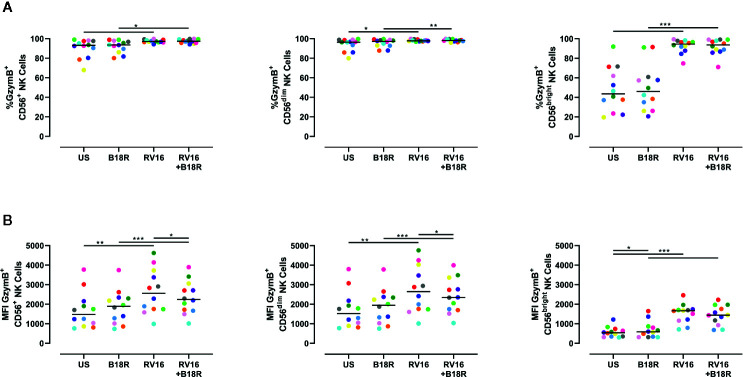
Blocking IFN-I signaling alters the amount of GzymB produced, but has little effect on the proportion of NK cells producing GzymB. PBMCs (n=12) were cultured *in vitro* with B18R (100 ng/ml) for 1 h, prior to stimulation with RV16 (MOI = 1), alongside an unstimulated control (US) for 24 h. **(A)** Percentage of GzymB-producing CD56^+^ (left), CD56^dim^ (middle), and CD56^bright^ (right) NK cells. **(B)** Amount (indicated by MFI) of intracellular GzymB in CD56^+^ (left), CD56^dim^ (middle), and CD56^bright^ (right) NK cells. Each colored symbol represents data from one donor, lines represent medians. Data are representative of three experiments. *p<0.05, **p<0.01, ***p<0.001 by Wilcoxon matched-pairs signed rank tests. IFN-I, type I interferon; GzymB, granzyme B; NK, natural killer; PBMC, peripheral blood mononuclear cell; UT, untreated; RV16, rhinovirus 16; MOI, multiplicity of infection; US, unstimulated; MFI, median fluorescence intensity.

The cytolytic granules released through directed exocytosis contain proteins, such as GzymB, which is important in NK cell-mediated apoptosis of target cells (Shresta et al., 1995). RV16 stimulation of PBMC cultures resulted in small, but statistically significant, increases in the proportion of CD56^+^ NK cells producing GzymB (**Figure 4A**, left). This was most apparent in the CD56^bright^ NK cell population (**Figure 4A**, right). Blocking of IFN-I signaling *in vitro* with B18R did not significantly alter the frequency of GzymB-producing NK cells (**Figure 4A**). RV16 stimulation also increased the intracellular GzymB MFI of CD56^+^ NK cells (**Figure 4B**, left), with changes observed in both the CD56^dim^ (**Figure 4B**, middle) and CD56^bright^ NK cell subsets (**Figure 4B**, right). Blocking of IFN-I signaling caused only minor changes in these responses.

### RV16 Induces IFNγ-Producing NK Cells in an IFN-I Dependent Manner

**Figure 5:**
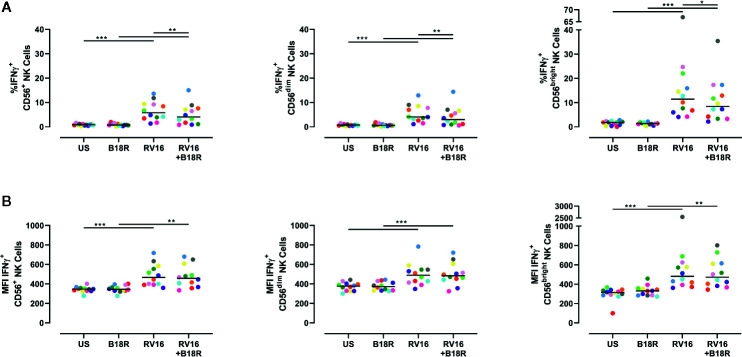
RV16 stimulation induces NK cell IFNγ production; this occurs to a lesser extent when IFN-I signaling is blocked. PBMCs from healthy people (n=12) were cultured *in vitro* with B18R (100 ng/ml) for 1 h, prior to stimulation with RV16 (MOI = 1), alongside an unstimulated control (US) for 24 h. **(A)** Percentage of IFNγ-producing CD56^+^ (left), CD56^dim^ (middle), and CD56^bright^ (right) NK cells. **(B)** Amount (indicated by MFI) of intracellular IFNγ in CD56^+^ (left), CD56^dim^ (middle), and CD56^bright^ (right) NK cells. Each colored symbol represents data from one donor, lines represent medians. Data are representative of three experiments. *p<0.05, **p<0.01, ***p<0.001 by Wilcoxon matched-pairs signed rank tests. IFN-I, type I interferon; NK, natural killer; IFNγ, interferon gamma; PBMC, peripheral blood mononuclear cell; UT, untreated; RV16, rhinovirus 16; MOI, multiplicity of infection; US, unstimulated; MFI, median fluorescence intensity.

NK cells are known to produce IFNγ in response to other viruses, including influenza viruses (Du et al., 2010). IFNγ activates multiple pathways associated with direct antiviral functions and immunoregulation, and promotes downstream protective immune responses (Biron and Brossay, 2001). Herein, we have found that RV16 stimulation resulted in a significant increase in the frequency of IFNγ-producing CD56^+^ NK cells (**Figure 5A**, left), and in the intracellular IFNγ MFI (**Figure 5B**, left). These trends were observed in both CD56^dim^ (**Figure 5A**, middle; **5B**, middle) and CD56^bright^ NK cell subsets (**Figure 5A**, right; **5B**, right). The increase in frequency of IFNγ-producing cells due to RV16 stimulation was most prominent in the CD56^bright^ NK cell subset (**Figure 5A**, right). When IFN-I was blocked, there was a significant decrease in the frequency of RV16-stimulated IFNγ-producing CD56^+^ NK cells, which was reflected in both the CD56^dim^ and CD56^bright^ NK cell subsets (**Figure 5A**). This indicated that the RV16-stimulated increase in the frequency of IFNγ-producing NK cells was only partially dependent on IFN-I signaling.

### IFN-I Signaling Is Involved in the RV16-Stimulated Release of GzymB and IFNγ

**Figure 6:**
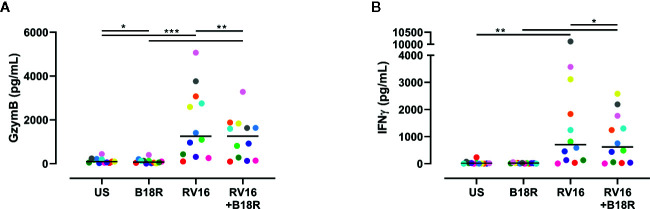
RV16 stimulated PBMCs release GzymB and IFNγ; this occurs to a lesser extent when IFN-I signaling is blocked. PBMCs from healthy people (n=12) were cultured *in vitro* with B18R (100 ng/ml) for 1 h, prior to stimulation with RV16 (MOI = 1), alongside an unstimulated control (US) for 24 h. ELISAs were performed on cell-free supernatants to determine GzymB and IFNγ concentrations. **(A)** Concentration of GzymB released into cell culture media by PBMCs. **(B)** Concentration of for IFNγ released into cell culture media by PBMCs. Each colored symbol represents data from one donor, lines represent medians. Data are representative of three experiments. *p<0.05, **p<0.01, ***p<0.001 by Wilcoxon matched-pairs signed rank tests. IFN-I, type I interferon; GzymB, granzyme B; IFNγ, interferon gamma; PBMC, peripheral blood mononuclear cell; RV16, rhinovirus 16; UT, untreated; MOI, multiplicity of infection; US, unstimulated; ELISA, enzyme-linked immunosorbent assay.

ELISA techniques were used to quantify RV16-stimulated GzymB and IFNγ release. Herein, we found that RV16 stimulation of PBMCs resulted in significant GzymB and IFNγ release into the culture supernatants (**Figure 6**). When IFN-I signaling was blocked, there was a decrease in the concentration of released GzymB, in both the presence and absence of RV16 stimulation (**Figure 6A**). In the presence of RV16 stimulation, blocking of IFN-I signaling also decreased IFNγ concentrations in culture supernatant (**Figure 6B**).

### NK Cells Make a Large Contribution to the Production of GzymB and IFNγ

As there are multiple cell types in PBMC (other than NK cells) that can respond to viral stimuli, the amount of GzymB and IFNγ in culture supernatant cannot be wholly attributed to NK cells (Hornung et al., 2002). Thus, we next determined the relative levels of GzymB and IFNγ produced by other cells types (T and NKT cells) versus NK cells. In order to do this, surface staining was used to identify T cells and NKT cells (**Figure S1**), and the iMFI was calculated for GzymB and IFNγ production for each of these cell types, as described by Darrah *et al.* (Darrah et al., 2007). We then scaled this relative to the size of each population by multiplying the iMFI by the frequency of each cell type of the total lymphocyte population.

**Figure 7:**
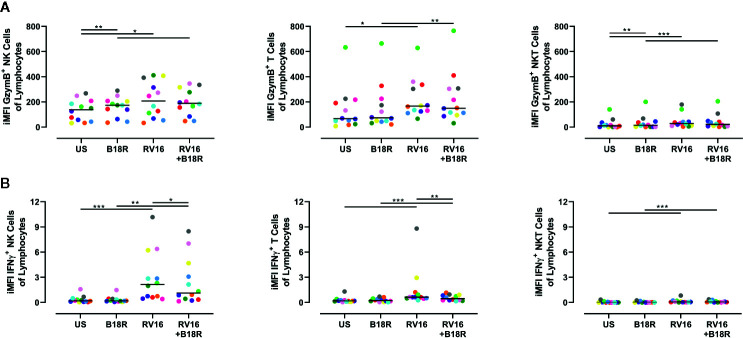
NK cells are responsible for producing a large amount of the GzymB and IFNγ seen in RV16-stimulated PBMCs. PBMCs from healthy people (n=12) were cultured *in vitro* with B18R (100 ng/ml) for 1 h, prior to stimulation with RV16 (MOI = 1), alongside an unstimulated control (US) for 24 h. Expression of cell surface markers and intracellular cytokine production was determined by flow cytometry. **(A)** iMFI GzymB-producing NK cells, T cells, and NKT cells, scaled to account for their population size. **(B)** iMFI IFNγ-producing NK cells, T cells, and NKT cells, scaled to account for their population size. Each colored symbol represents data from one donor, lines represent medians. Data are representative of three experiments. *p<0.05, **p<0.01, ***p<0.001 by Wilcoxon matched-pairs signed rank tests. NK, natural killer; GzymB, granzyme B; IFNγ, interferon gamma; RV16, rhinovirus 16; PBMC, peripheral blood mononuclear cell; IFN-I, type I interferon; UT, untreated; MOI, multiplicity of infection; US, unstimulated; iMFI, integrated median fluorescence intensity; NKT, natural killer T.

We found that RV16 stimulation significantly upregulated the relative iMFI of GzymB^+^ cells in NK cells, T cells, and NKT cells, both in the presence and absence of IFN-I signaling (**Figure 7A**). In the NK cell and NKT cell populations, the relative iMFI of GzymB^+^ cells increased when IFN-I signaling was blocked in the absence of RV16 stimulation (**Figure 7A**, left and right). In all the conditions tested, the relative iMFI of GzymB^+^ cells was highest in NK cells (**Figure 7A**, left). It is worth noting that, unlike in the ELISA results, there was no significant decrease in RV16-stimulated GzymB when IFN-I signaling was blocked.

RV16 stimulation significantly upregulated the relative iMFI of IFNγ^+^ cells in all three of the populations tested, in both the presence and absence of IFN-I signaling (**Figure 7B**). In RV16-stimulated cultures, blocking of IFN-I signaling resulted in a significant decrease in the relative iMFI of IFNγ^+^ NK cells and T cells, but not NKT cells (**Figure 7B**). The trends observed in NK cells and T cells reflect the levels of IFNγ detected in the culture supernatant (**Figure 7B**, left and middle). Stimulation with RV16 caused the largest change in the relative iMFI of IFNγ^+^ cells in the NK cell population (**Figure 7B**, left).

## Discussion

This study aimed to investigate the possible role for NK cells in the immune response to RV infection and determine the extent to which RV16-induced activation and function of NK cells *in vitro* is dependent on IFN-I signaling. The key findings to emerge were that in cultured PBMCs from healthy people, RV16 stimulation affected NK cell activation and function, in a manner that was partially regulated by IFN-I signaling. IFN-I signaling partly contributed to RV-stimulated NK cell activation. Blocking of IFN-I signaling in PBMC cultures prior to RV16 stimulation reduced, but did not eliminate, NK cell activation. The magnitude of the effect varied between donors, but was generally modest.

IFN-I has previously been shown to play both a direct and indirect role in the activation of NK cells (Biron et al., 1999; Cooper et al., 2001b; Hansbro et al., 2008). RV16 induces PBMCs to release IFN-I into the supernatant within 24 h (Khaitov et al., 2009). DC-mediated activation of NK cells involves both IFN-I dependent and independent mechanisms (Benlahrech et al., 2009). This is consistent with the NK cell activation observed in this study.

NK cells can be activated by several stimuli, including interactions with APCs, and cytokines, including IL-2, IL-12, IL-15, and IL-18 (Orange and Biron, 1996a; Biron et al., 1999; Biron and Brossay, 2001; Cooper et al., 2001b; Moretta, 2002; He et al., 2004; Schoenborn and Wilson, 2007; Vivier et al., 2008). RV infection induces IL-15 expression from DCs and BECs, and can activate NK cells, inducing IFNγ production, independent of IFN-I signaling (Jayaraman et al., 2014; Xi et al., 2017; Kronstad et al., 2018). RV16-stimulated activation of NK cells in PBMC cultures where IFN-I signaling is blocked, suggests that RV16 can activate NK cells *via* IFN-I-independent mechanisms, similar to what has been reported for other viruses (Gary-Gouy et al., 2002; Hornung et al., 2002). Further experiments are required to analyze the exact nature of these IFN-I-independent mechanisms involved in RV16-stimulated NK cell activation.

Some respiratory viruses, such as influenza, can directly interact with NK cells to elicit immune responses (Ennis et al., 1981; Sirén et al., 2004; Hwang et al., 2013). The use of PBMC cultures in this study did not allow us to determine the contribution of direct interactions between RV16 and NK cells, nor whether RV16 activates NK cells indirectly *via* other cells, such as APCs. Future experiments could determine this by studying purified NK cells.

RV16-stimulated NK cell degranulation was reduced when IFN-I signaling was blocked *in vitro*, as shown in **Figure 3**. This correlates with previous research into murine models of viral infection, with IFN-I signaling shown to contribute to the degranulation of NK cells during MCMV infection (Orange and Biron, 1996b; Nguyen et al., 2002). Interestingly, blocking IFN-I signaling reduced the proportion of CD107a^+^ NK cells both in virus-stimulated and unstimulated cultures. The latter observation may be attributed to autologous DCs inducing NK cell degranulation, as described by others (Hornung et al., 2002; Walwyn-Brown et al., 2018). Alternatively, it is possible there is a certain amount of constitutive IFN-I signaling that produces low-level NK cell degranulation. It has been suggested that CD107a protects degranulating cells from their own cytolytic granules, providing a basis for constitutive CD107a expression (Cohnen et al., 2013). There were no notable differences between the degranulation of CD56^dim^ and CD56^bright^ NK cell subsets.

GzymB is a cytotoxic mediator released from NK cells to cause lysis of target cells (Fehniger et al., 2007). While RV16 stimulation increased the frequency of GzymB-producing NK cells, blocking of IFN-I signaling did not have a significant impact. Despite the frequency of GzymB-producing NK cells not changing significantly, the amount of intracellular GzymB (which was significantly increased in response to viral stimulation) was lower in the absence of IFN-I signaling. Previous studies have observed similar trends in murine vaccinia virus infection, where the addition of IFN-I directly stimulated NK cell production of GzymB (Martinez et al., 2008). While constitutive expression of GzymB was noticeably higher in CD56^dim^ NK cells, similar trends were still observed in CD56^bright^ NK cells. RV16 stimulation induced GzymB release into culture supernatant, in a manner partially dependent on IFN-I signaling. Importantly, NK cells were observed to produce more GzymB than T cells or NKT cells.

Stimulation by viruses and cytokines induces NK cell production of IFNγ (Cooper et al., 2001b; Papadopoulos et al., 2002b; Maroof et al., 2008). RV16 stimulation significantly increased both the frequency of IFNγ-producing NK cells and their level of intracellular IFNγ. Blocking IFN-I signaling resulted in a reduction in the frequency of IFNγ-producing NK cells in RV16-stimulated cultures. This indicates that IFN-I signaling plays a role in RV16-stimulated IFNγ production by NK cells, and is consistent with previous studies on the role of IFN-I on NK cell IFNγ in other viral infections (Biron et al., 1999; Gary-Gouy et al., 2002; Martinez et al., 2008; Kronstad et al., 2018). Notably, almost three times as many CD56^bright^ NK cells were producing IFNγ in response to RV16 stimulation, than CD56^dim^ NK cells. RV16 stimulation also significantly increased the amount of released IFNγ, and this was also partly dependent on IFN-I signaling. Importantly, NK cells were found to be more responsive to both RV16 stimulation and IFN-signaling, than T cells or NKT cells. Subsequent studies could elucidate the variations in NK cell surface receptor repertoire, specifically the density of IFNAR, that may contribute to the differential effects of RV on the CD56^dim^ and CD56^bright^ NK cell subsets (Cooper et al., 2001a; Sedlmayr et al., 2004).

The limitations of this study must be acknowledged. Firstly, the use of PBMCs as opposed to lung immune cells for *in vitro* experiments. However, NK cells in the lung seem to primarily consist of circulating rather than tissue resident cells (Marquardt et al., 2017). In addition to this, RV stimulation of PBMCs has been shown to be a suitable *in vitro* model in which to observe immune responses (Message and Johnston, 2001; Papadopoulos et al., 2002b; Hornung et al., 2002; Xi et al., 2015; Xi et al., 2017). It is also worth noting that the sample size of this study was small (n=12); despite this, there was enough statistical power identify significant differences between groups. The age of participants in this study (21.6 ± 2.8 years) was also restricted. Future studies should be conducted in larger cohorts, with a broader age range, to confirm these findings and account for interindividual variation in response to viral stimulation. This study only assessed NK cell response at a single time point with a single MOI of one serotype of human RV. There are over 150 serotypes of human RV (Glanville and Johnston, 2015). These serotypes are categorized into major and minor subtypes, based on their method of cell entry; however, even serotypes that share a common method of cell entry can follow different endocytic pathways and release of viral genome at different locations within the infected cells (Blaas and Fuchs, 2016), leading to diversity in the elicited immune response (Wark et al., 2009). Despite this, recognition of pathogen associated molecular patterns that are highly conserved across RV serotypes, such as ssRNA, by pattern recognition receptors triggers the activation of the innate immune response (Triantafilou et al., 2011). Activation of PBMCs in response to RV stimulation has also been shown to be dose-dependent (Gern et al., 1996). Future studies using multiple MOIs and different serotypes of RV could address the question of dose-dependent and serotype-dependent differences in NK cell responses. It is also important to acknowledge that immune cells, including NK cells, do not interact with RV in circulation, but at the airway epithelium. Thus, further studies should be conducted in co-cultures of respiratory epithelial cells and NK cells, in order to observe the NK cell response to virally infected respiratory epithelial cells. Future studies should also assess the levels of RV-specific neutralizing antibodies in the serum of each participant. A deficiency in RV-specific neutralizing IgG antibodies, specifically those targeting the VP1 viral capsid protein, has previously been associated with increased risk of exacerbations in patients with COPD (Yerkovich et al., 2012). Future studies should examine whether variations in RV-induced NK cell activation are associated with susceptibility to colds. A study of this nature would require a large cohort of participants to have sufficient study power.

In conclusion, we demonstrated that RV16 stimulates NK cells *in vitro*, and that this response is partially, but not completely, regulated by IFN-I signaling. These results established that RV16 stimulation of PBMCs leads to NK cell activation, degranulation, cytotoxic mediator production, cytokine production, and the release of cytotoxic mediators and cytokines into the culture supernatant. These aspects of NK cell activation and function were all partially dependent on IFN-I signaling. While deficient IFN-I signaling may play some role in the susceptibility of asthmatics to more persistent and severe infections, our findings also indicate that further studies need to examine other cytokines and APC function, and how this impacts on NK cell function. This study provides an important foundation for future studies into NK cell activation and function in asthma.

## Data Availability Statement

The datasets generated for this study are available on request to the corresponding author.

## Ethics Statements

The studies involving human participants were reviewed and approved by The University of Queensland Human Research Ethics Committee and Metro South Human Research Ethics Committee. The patients/participants provided their written informed consent to participate in this study.

## Author Contributions

SH and JU contributed to the conception and design of the study. SH and YX conducted the experiments and statistical analysis. SH wrote the manuscript. YX and JU contributed to the revision of the manuscript and approved the submitted version. All authors contributed to the article and approved the submitted version.

## Funding

The National Health and Medical Research Council (NHMRC) of Australia supported this work *via* Project grant APP1128010.

## Conflict of Interest

The authors declare that the research was conducted in the absence of any commercial or financial relationships that could be construed as a potential conflict of interest.
