# Supplementary material for: Natural Killer Cells and Host Defense Against Human Rhinoviruses Is Partially Dependent on Type I IFN Signaling
Source: Front Cell Infect Microbiol. 2020 Oct 21;10:510619. doi: 10.3389/fcimb.2020.510619 (PMC7609819; doi:10.3389/fcimb.2020.510619)
Supplement: Supplementary file 1 [file Table_1.docx]

| **Table S1**. Monoclonal antibodies used for flow cytometry experiments | | | | | |
| --- | --- | --- | --- | --- | --- |
| ***Marker*** | ***Fluorochrome*** | ***Clone*** | ***Catalogue #*** | ***Company*** | ***Dilution*** |
| CD3 | FITC | UCHT1 | 555332 | BD Pharmingen | 1:10 |
| CD14 | PerCp-Cy5.5 | M5E2 | 301824 | BioLegend | 1:100 |
| CD19 | APC | HIB19 | 555415 | BD Pharmingen | 1:100 |
| CD56 | PE-Cy7 | CMSSB | 25-0567-42 | eBioscience | 1:10 |
| CD69 | APC-Cy7 | FN50 | 557756 | BD Horizon | 1:10 |
| CD107a | BV786 | H4A3 | 563869 | BD Horizon | 1:50 |
| IFNγ | PE | B27 | 559327 | BD Pharmingen | 1:5 |
| Granzyme B | BV421 | GB11 | 563389 | BD Horizon | 1:5 |

**
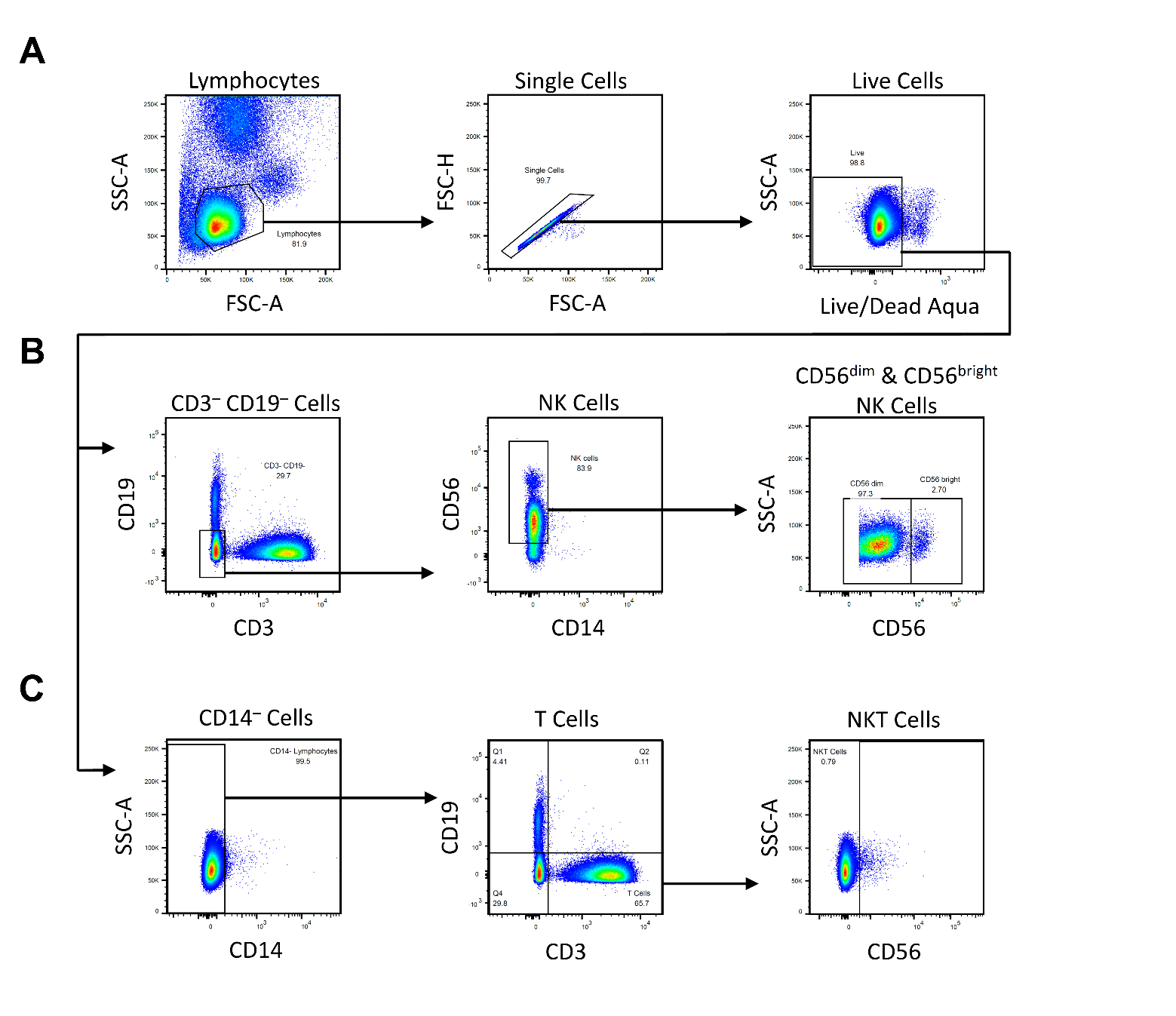
Figure S1**. Gating strategy used to identify NK cells, T cells, and NKT cells. PBMCs from healthy people (n=12) were cultured and stained for analysis by flow cytometry. **(A)** Total lymphocytes were gated based on SSC and FSC morphology, from which doublets and dead cells (Live/Dead Aqua^+^) were excluded. **(B)** T cells (CD3^+^), B cells (CD19^+^), and monocytes (CD14^+^), were excluded, and NK cells (CD56^+^) were identified from the CD3^–^CD19^–^CD14^–^ population. CD56^dim^ and CD56^bright^ populations were identified and gated from the total CD56^+^ population. **(C)** Monocytes (CD14^+^) were excluded and T cells (CD3^+^) and NKT cells (CD3^+^CD56^+^) were gated. NK, natural killer; NKT, natural killer T; PBMC, peripheral blood mononuclear cell; SSC-A, side scatter-area; FSC-A, forward scatter-area; FSC-H, forward scatter-height.
